# Clinical and Microbiological Factors Associated with High Nasopharyngeal Pneumococcal Density in Patients with Pneumococcal Pneumonia

**DOI:** 10.1371/journal.pone.0140112

**Published:** 2015-10-14

**Authors:** Helena Alpkvist, Simon Athlin, Pontus Nauclér, Björn Herrmann, Guma Abdeldaim, Hans-Christian Slotved, Jonas Hedlund, Kristoffer Strålin

**Affiliations:** 1 Department of Infectious Diseases, Karolinska University Hospital, Stockholm, Sweden; 2 Unit of Infectious Diseases, Department of Medicine Huddinge, Karolinska Institutet, Stockholm, Sweden; 3 Department of Infectious Diseases, Faculty of Medicine and Health, Örebro University, Örebro, Sweden; 4 Unit of Infectious Diseases, Department of Medicine Solna, Karolinska Institutet, Stockholm, Sweden; 5 Section of Clinical Bacteriology, Department of Medical Sciences, Uppsala University, Uppsala, Sweden; 6 Department of Medical Microbiology and Parasitology, Faculty of Medicine, Benghazi University, Benghazi, Libya; 7 Department of Microbiology and Infection Control, Statens Serum Institut, Copenhagen, Denmark; Rockefeller University, UNITED STATES

## Abstract

**Background:**

We aimed to study if certain clinical and/or microbiological factors are associated with a high nasopharyngeal (NP) density of *Streptococcus pneumoniae* in pneumococcal pneumonia. In addition, we aimed to study if a high NP pneumococcal density could be useful to detect severe pneumococcal pneumonia.

**Methods:**

Adult patients hospitalized for radiologically confirmed community-acquired pneumonia were included in a prospective study. NP aspirates were collected at admission and were subjected to quantitative PCR for pneumococcal DNA (Spn9802 DNA). Patients were considered to have pneumococcal etiology if *S*. *pneumoniae* was detected in blood culture and/or culture of respiratory secretions and/or urinary antigen test.

**Results:**

Of 166 included patients, 68 patients had pneumococcal DNA detected in NP aspirate. Pneumococcal etiology was noted in 57 patients (84%) with positive and 8 patients (8.2%) with negative test for pneumococcal DNA (*p*<0.0001). The median NP pneumococcal density of DNA positive patients with pneumococcal etiology was 6.83 log_10_ DNA copies/mL (range 1.79–9.50). In a multivariate analysis of patients with pneumococcal etiology, a high pneumococcal density was independently associated with severe pneumonia (Pneumonia Severity Index risk class IV-V), symptom duration ≥2 days prior to admission, and a medium/high serum immunoglobulin titer against the patient’s own pneumococcal serotype. NP pneumococcal density was not associated with sex, age, smoking, co-morbidity, viral co-infection, pneumococcal serotype, or bacteremia. Severe pneumococcal pneumonia was noted in 28 study patients. When we studied the performance of PCR with different DNA cut-off levels for detection of severe pneumococcal pneumonia, we found sensitivities of 54–82% and positive predictive values of 37–56%, indicating suboptimal performance.

**Conclusions:**

Pneumonia severity, symptom duration ≥2 days, and a medium/high serum immunoglobulin titer against the patient’s own serotype were independently associated with a high NP pneumococcal density. NP pneumococcal density has limited value for detection of severe pneumococcal pneumonia.

## Introduction

*Streptococcus pneumoniae* is the most common microbiological cause of community-acquired pneumonia (CAP) [1]. In a recently published meta-analysis [2] it was found to be the etiology in 27.3% of adult CAP cases.

The drug of choice to treat pneumococcal pneumonia, narrow-spectrum penicillin [3], can be targeted if pneumococcal etiology is established. Recently, the Infectious Diseases Society of America [4] encouraged development of new and rapid tests, e.g. polymerase chain reaction (PCR) tests, to facilitate pathogen-directed therapy in CAP, and to identify patients with severe disease.

PCR for *S*. *pneumoniae* applied to blood samples has shown suboptimal performance [5]. However, detection of *S*. *pneumoniae* DNA in nasopharyngeal (NP) secretions is a promising concept, since most patients with pneumococcal pneumonia are colonized with *S*. *pneumoniae* in the nasopharynx [6, 7], and since NP secretions can be easily obtained. With a quantitative PCR for the pneumococcal gene *lytA*, Albrich et al. [8] found that a pneumococcal density of ≥8000 DNA copies /mL in NP secretions could distinguish pneumococcal pneumonia from asymptomatic colonization, with a sensitivity of 82.2% and a specificity of 92.0%. Accordingly, with a quantitative PCR for the pneumococcal gene fragment Spn9802 on NP aspirate, at cut-off 10^4^ DNA copies/mL, our group found positivity rates of 84% in CAP patients with NP culture positive for *S*. *pneumoniae*, 6.0% in CAP patients with NP culture negative for *S*. *pneumoniae*, and 3.6% in asymptomatic controls [9].

Albrich et al. [10] recently found that NP pneumococcal density correlated with mortality, in a patient cohort with HIV infection. Apart from that study, very little information is available on the correlation between NP pneumococcal density and clinical characteristics in pneumococcal pneumonia patients.

In order to improve our understanding about NP pneumococcal density in pneumococcal pneumonia, we aimed to study if certain clinical characteristics and/or microbiological factors are associated with a high NP pneumococcal density, in a cohort of well characterized CAP patients. Secondly, we aimed to study if a high NP pneumococcal density is useful for detection of severe pneumococcal pneumonia.

## Materials and Methods

### Patients

During a 2.5-year period (November 1999—April 2002), 235 patients hospitalized for CAP at the Department of Infectious Diseases, Örebro University Hospital, Örebro, Sweden, were enrolled in a prospective study [11]. Criteria for CAP were acute illness, radiological signs of pulmonary consolidation and at least two of five signs or symptoms (fever of >38°C, dyspnea, cough, pleuritic chest pain and abnormal lung auscultation), and no hospitalization during the preceding month. The scoring system of Fine et al. [12] was used for calculating Pneumonia Severity Index (PSI) at admission. Patients with PSI risk class IV-V were considered to have severe CAP. At study enrollment, the patient was specifically asked about onset of illness, in order to obtain accurate information about symptom duration prior to admission. In addition to symptom duration, we prospectively collected data on vital parameters, smoking, and antibiotic therapy prior to admission. Data on co-morbidities (solid tumor, blood malignancy, liver disease, renal disease, chronic obstructive pulmonary disease, heart disease, stroke and diabetes) was collected from the records. Mortality data was obtained from the Swedish Population Register.

We did not collect any vaccination data. However, the usage of the 23-valent pneumococcal polysaccharide vaccine was very low in the Örebro County during the study period (data not shown).

**Fig 1:**
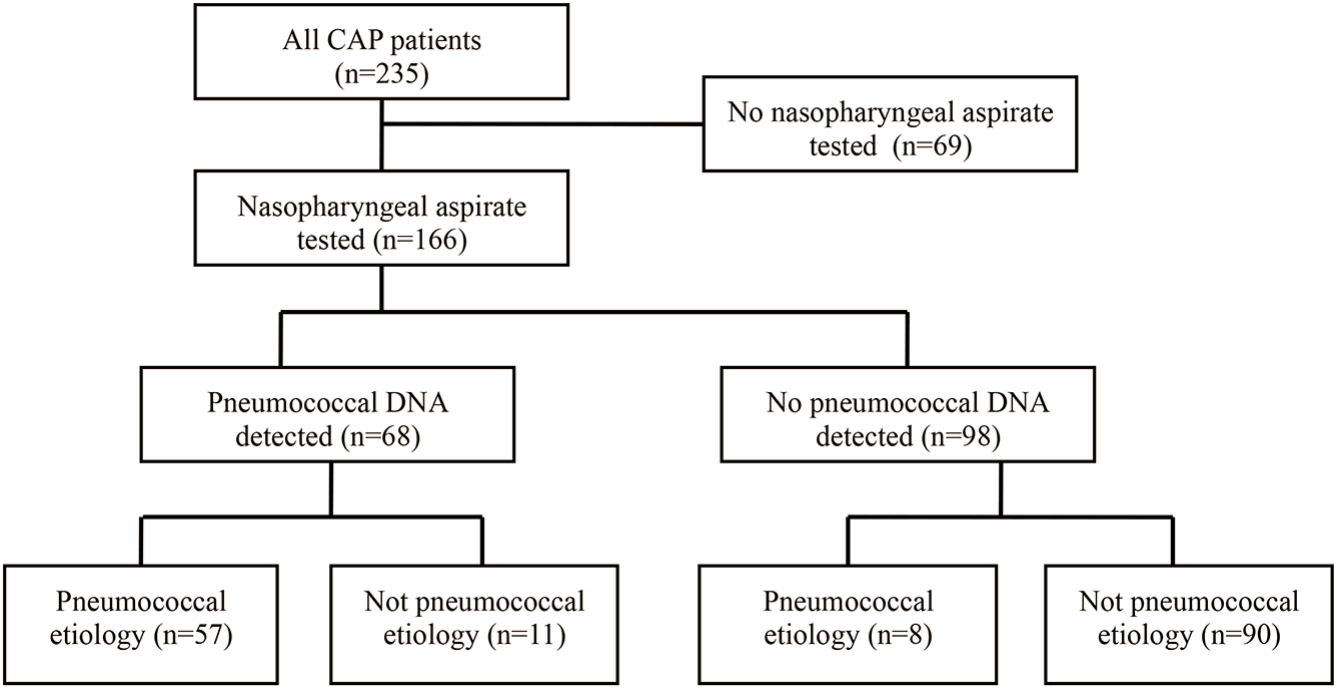
Flow chart of the study population with and without nasopharyngeal aspirate tested with PCR for pneumococcal DNA.

An NP aspirate sample, collected at admission, was available for the study in 166 cases (Fig 1). Among 69 cases with no NP aspirate available, 15 cases had no NP aspirate collected and the remaining 54 cases had no NP aspirate sample left for the study. The 166 patients included (47% female) and 69 patients not included (49% female) had similar median age (72 years, range 18–96 years; and 70 years, range 25–94 years) and similar median PSI score (85, range 18–191; and 80, range 31–144). Pneumococcal bacteremia was noted in 9.0% (15/166) of the included cases and in 14% (10/69) of the non-included cases (*p* = 0.32).

The 166 samples were analyzed with quantitative real-time PCR for the *S*. *pneumoniae* specific DNA fragment Spn9802. In a previous publication [9] these PCR results and other pneumococcal PCR results were compared with results of culture on NP aspirate, but not with any other microbiological results or clinical data. Sixty-eight patients were positive and 98 patients were negative for pneumococcal DNA in NP aspirate. NP pneumococcal densities (log_10_ DNA copies/mL) were determined in the DNA positive cases.

### Clinical samples and conventional microbiological methods

NP aspirate samples were collected with a catheter connected to an electronic suction device. After aspiration, 0.5 to 1 mL of NaCl (0.85%) was aspirated to collect the secretions within the catheter. Sputum samples with >5 neutrophils per squamous epithelial cell were considered to be representative for the lower respiratory tract, and were cultured according to the method described by Kalin et al. [13]. The detection limit for a positive sputum culture was 10^5^colony-forming units/mL. A representative sputum sample was collected at admission in 82 study patients (49%). The sputum samples and NP aspirates were cultured on blood agar and hematin agar, with incubation in carbon dioxide for 24–48 h [9]. Bacterial pathogens were identified according to standard microbiological methods [14].

Blood cultures were collected in all study patients, and were analyzed with a Bactec^TM^ non-radiometric system (Becton Dickinson, USA).

The BinaxNOW^®^ *Streptococcus pneumoniae* urinary antigen test (Alere Inc., USA) was analyzed on non-concentrated urine in 151 study patients (91%), according to the instructions of the manufacturer.

Serology on paired sera (acute serum and convalescent serum after approximately 4 weeks) for detection of influenza A virus, influenza B virus and respiratory syncytial virus, was run in 149 study patients (90%). We used complement fixation technique, with serology titers of 1:10, 1:20, 1:40, 1:80, 1:160 and 1:320. The patient was considered to have a viral co-infection if a ≥4-fold titer increase between paired sera was noted.

### Definition of pneumococcal etiology

A patient was considered to have pneumococcal etiology if *S*. *pneumoniae* was detected by blood culture and/or culture of respiratory secretions and/or urinary antigen test. If none of these diagnostic method was positive for *S*. *pneumoniae*, the patients was considered not to have pneumococcal etiology.

### Pneumococcal serotypes

All pneumococcal isolates identified from culture of blood, sputum, and NP secretions, 0–3 isolates per patient, were serotyped by using the Quellung reaction [15].

The serotypes were divided into two groups according to degree of encapsulation, based on studies of Weinberger et al. [16, 17]. Serotypes with a mean area of ≥500 pixels for the zone of FICT-dextran were considered to have a high degree of encapsulation (serotypes 3, 6B, 11A, 12F, 19A, 19F, 23F and 35B) and serotypes with <500 pixels were considered to have a low degree of encapsulation (serotypes 1, 4, 7F, 9N, 9V and 14). We chose to divide serotypes this way, since serotypes with high and low degree of encapsulation have been associated with different disease severity [16] and different ability to induce an antibody response [18].

### Immunoglobulin antibodies

The total serum immunoglobulin (S-Ig) level against the patient´s own pneumococcal serotype in acute serum was analyzed with an enzyme-linked immunosorbent assay (ELISA)[19], at Statens Serum Institut, Copenhagen, Denmark, in 2003. The principles and limitations of the ELISA procedure have been described in detail in two recent publications [18, 20]. The method was limited to the 23 pneumococcal serotypes included in the 23-valent polysaccharide vaccine, i.e. serotypes 1, 2, 3, 4, 5, 6B, 7F, 8, 9N, 9V, 10A, 11A, 12F, 14, 15B, 17F, 18C, 19A, 19F, 20, 22F, 23F, 33F. Briefly, polysaccharides from the American Type Culture Collection were used for coating. All serum samples were adsorbed by adding cell-wall polysaccharides (CWPS) to inhibit non-specific binding [21]. An in-house standard serum, which correlated with the international standard serum 89SF [22] was used. On each ELISA plate, a 2-fold dilution series from 1:200 to 1:25600 of the standard serum was added. For each patient sample, a dilution series of 1:100, 1:300, 1:900 and 1:2700 was made. By comparing the optical density data from the dilution series of the patient serum with standard serum, an antibody titer in arbitrary units (AU) was achieved [19].

The World Health Organization Collaborating Center for Reference and Research on Pneumococci at the Statens Serum Institut previously used this ELISA in a program for evaluation of vaccine effectiveness and as a guide for pneumococcal revaccination with the 23-valent pneumococcal polysaccharide vaccine [19, 20]. In that previous program, ELISA for six standardized serotypes was run and a geometric mean (GM) of the ELISA titers was calculated. Revaccination was recommended if the GM titer was <25 AU, or <40 AU with more than one of the six type-specific antibody levels being <25 AU. A GM titer of >40 AU was considered to be protective [19].

In the present study, the admission S-Ig titer against the patient´s own pneumococcal serotype was determined in 47 patients with pneumococcal etiology and *S*. *pneumoniae* DNA detected in NP aspirate. We considered an S-Ig titer of ≤25 AU as a low S-Ig titer and an S-Ig titer of >25 AU as a medium/high S-Ig titer.

We have recently presented S-Ig results for different serotypes and clinical presentations, from the present overall study [18].

### DNA extraction and Spn9802 PCR

DNA from NP aspirates was extracted using the Qiamp DNA mini kit (QIAGEN, Germany). PCR for Spn9802 DNA was performed in a Rotor-Gene 3000 instrument (QIAGEN), at the Department of Clinical Microbiology, Uppsala University Hospital, Uppsala, Sweden. The primers used were Spn9802-F (5’-AGTCGTTCCAAGGTAACAAGTCT-3’) and Spn9802-R (5’-ACCAACTCGACCACCTCTTT-3’). For detection, the Spn9802-FAM probe was designed as 5’-FAM-aTcAGaTTgAAGCTgAtaAAaCgA-black hole quencher 1 (BHQ1)-3’, lower case letters indicating locked nucleic acids. The PCR method is further described in a previous article [9], although in that article the probe sequence is incorrectly described (corrected in [23]).

### Statistical analysis

Pearson’s chi-square test or Fisher’s exact test were used for comparisons of proportions. Kruskal-Wallis nonparametric analysis of variance was used for comparison of medians of S-Ig titer subgroups. Mann-Whitney U test was used for comparisons of medians of NP pneumococcal densities in univariate analyses. A multivariate regression analysis was performed with bootstrapping to obtain confidence intervals. Variables that were associated with NP pneumococcal density in univariate analyses (*p*-values <0.1) were included in the multivariate model. A *p-*value below 0.05 was regarded as significant.

### Ethics Statement

The study was approved by the Ethics Committee of Örebro County Council (868–1999). All included patients provided their verbal informed consent. The physician who included the patient signed in the case report form that all inclusion criteria were met, which included verbal informed consent. This informed consent procedure was in agreement with routine practice for observational studies in Sweden during the study period, and it was approved by the Ethics Committee.

## Results

### Study population

Fig 1 presents a flow chart of the 166 study patients, including 68 patients with positive and 98 patients with negative NP PCR test for pneumococcal DNA. Pneumococcal etiology was noted in 57 (84%) of the DNA positive cases and in 8 (8.2%) of the DNA negative cases (*p*<0.0001).

Table 1 shows demographic data of the study subjects. Compared with patients with negative DNA test, patients with positive DNA test tended to be smokers more often, 60% (41/68) versus 44% (43/98; *p* = 0.054), and to have viral co-infection more often, 24% (16/68) versus 12% (12/98; *p* = 0.089). Any co-morbidity was noted more often in patients with pneumococcal etiology (62%, 40/65) than in patients without pneumococcal etiology (42%, 42/101; *p* = 0.019). Antibiotic treatment prior to admission was noted in only one patient with positive DNA test, a 23 year old man with pneumococcal etiology, who had received penicillin V. However, among CAP patients without pneumococcal etiology, with negative DNA test, 27% (24/90) had received antiobitics prior to admission. They had received penicillin V (n = 12), amoxicillin (n = 3), amoxicillin-clavulanate (n = 1), amoxicillin & clarithromycin (n = 1), cefadroxil (n = 1), erythromycin (n = 1), doxycycline (n = 2), clindamycin (n = 1), ciprofloxacin (n = 1), and trimethoprim (n = 1).

**Table 1:** Characteristics of patients with community-acquired pneumonia with pneumococcal DNA detected and not detected in nasopharyngeal (NP) aspirate.

Characteristics		Pneumococcal DNA in NP aspirate		No pneumococcal DNA in NP aspirate			
	All patients (N = 166)	Pneumo-coccal etiology ^a^(N = 57)	Not pneumo-coccal etiology (N = 11)	Pneumo-coccal etiology ^a^(N = 8)	Not pneumo-coccal etiology (N = 90)	*P*-valuePneumo-coccal DNA+ *vs* Pneumo-coccalDNA-	*P*-value Pneumo-coccal etiology *vs* Not pneumo-coccal etiology
Female gender	78 (47)	27 (47)	4 (36)	4 (50)	43 (48)	0.89	0.88
Age, median (range) years	72 (18–96)	69 (23–96)	70 (39–91)	69 (31–92)	74 (18–93)	0.77	0.71
Any co-morbidity ^b^	82 (49)	33 (58)	4 (36)	7 (88)	38 (42)	0.36	**0.019**
Smoking, current or previous	84 (51)	35 (61)	6 (55)	2 (25)	41 (46)	0.054	0.25
Antibiotics prior to admission	25 (15)	1 (1.8)	0	0	24 (27)	**<0.0001**	**<0.0001**
Symptoms ≥2 days prior to admission	121 (73)	38 (67)	8 (73)	5 (62)	70 (78)	0.28	0.17
Viral co-infection	28 (17)	14 (25)	2 (18)	0	12 (13)	0.089	0.28
Bilateral chest X-ray infiltrates	46 (28)	16 (28)	0	0	30 (33)	0.41	0.59
Pneumonia severity index, median (range)	85 (18–191)	88 (31–191)	65 (41–140)	94 (31–132)	83 (18–179)	0.53	0.25
Pneumonia severity index, risk class IV-V	68 (41)	23 (40)	4 (36)	5 (62)	36 (40)	0.91	0.78
Intensive care unit admission	9 (5.4)	3 (5.3)	1 (9.1)	2 (25)	3 (3.3)	1.0	0.32
30-day mortality	6 (3.6)	1 (1.8)	1 (9.1)	0	4 (4.4)	1.0	0.41

Data are presented as numbers (%), unless otherwise indicated.

^a^ *Streptococcus pneumoniae* detected by blood culture and/or culture of respiratory secretions and/or urinary antigen test.

^b^ Solid tumor, blood malignancy, liver disease, renal disease, chronic obstructive pulmonary disease, heart disease, stroke, diabetes.

### Microbiological findings in patients with pneumococcal etiology

Pneumococcal DNA was detected in NP aspirate in 57 patients with pneumococcal etiology, with *S*. *pneumoniae* identified by blood culture (n = 12), urinary antigen test (n = 33), and culture of respiratory secretions (n = 53). Fifty-four of these patients were positive for *S*. *pneumoniae* in at least one culture. Serotyping was performed on pneumococcal isolates from blood, sputum, and NP secretions in 8 patients, blood and NP secretions in 3 patients, blood alone in one patient, sputum and NP secretions in 21 patients, sputum alone in 2 patients, and NP secretions alone in 19 patients. In patients with isolates from different culture sites, the same serotype was noted in all cases but one, a patient with serotype 35A in sputum and 35B in NP secretions. The following pneumococcal serotypes were noted: serotypes 14 (n = 9), 23F (n = 8), 3 (n = 7), 7F (n = 5), 11A (n = 4), 4 (n = 3), 1 (n = 2), 9N (n = 2), 9V (n = 2), 19A (n = 2), 19F (n = 2), 2 (n = 1), 6B (n = 1), 12F (n = 1), 16F (n = 1), 22F (n = 1), 35A & 35B (n = 1), 38 (n = 1), and 42 (n = 1). Co-infection with viral pathogens was noted in 14 cases (25%), i.e. influenza A (n = 9), influenza B (n = 2), and respiratory syncytial virus (n = 3).

In the 8 patients with pneumococcal etiology and negative DNA test, *S*. *pneumoniae* was identified by blood culture (n = 3), urinary antigen test (n = 4), and culture of respiratory secretions (n = 3). Six patients were culture positive for *S*. *pneumoniae* in only one culture each, and serotyping of the 6 isolates showed 6 different serotypes, i.e. serotypes 3, 7F, 19A, 22F, 23F, and 31. The remaining 2 patients were positive for *S*. *pneumoniae* only by urinary antigen test. No viral co-infection was noted in this group.

### Serum-immunoglobulin titers at admission

The admission S-Ig titer against the patient´s own serotype was determined in 47 patients with pneumococcal etiology, with the serotype included in the 23-valent polysaccharide vaccine, and *S*. *pneumoniae* DNA detected in NP aspirate. Thus, we determined S-Ig for one serotype per patient, i.e. for serotypes 14 (n = 9), 23F (n = 7), 3 (n = 7), 7F (n = 5), 11A (n = 4), 4 (n = 2), 9N (n = 2), 9V (n = 2), 19A (n = 2), 19F (n = 2), 1 (n = 1), 2 (n = 1), 6B (n = 1), 12F (n = 1), and 22F (n = 1). Three other DNA positive patients with pneumococcal etiology and serotypes suitable for the ELISA method (serotypes 1, 4, and 23F) were not subjected to serology, as they did not have a serum sample from admission available.

Among 47 patients, the median S-Ig titer was 38 AU (range, 5–243 AU). The S-Ig titer was ≤25 AU in 17 patients (36%) and >25 AU in 30 patients (64%). The median S-Ig titer was not significantly different between patients with symptom durations prior to admission of <2 days (42 AU; range, 12–150 AU; n = 17), 2–6 days (25 AU; range, 5–161 AU; n = 22), and >6 days (83 AU; range, 15–243 AU; n = 8; *p* = 0.09).

### Nasopharyngeal pneumococcal densities

In 68 patients with pneumococcal DNA detected in NP aspirate, the median NP pneumococcal density was 6.51 (range, 1.79–9.50) log_10_ DNA copies/mL. The NP pneumococcal density was higher in patients with pneumococcal etiology than in patients without pneumococcal etiology, median 6.83 (range, 1.79–9.50) vs. 4.98 (range, 3.28–6.49) log_10_ DNA copies/mL, *p*<0.001. However, the median NP pneumococcal densities were equal among patients with pneumococcal etiology with positive and negative *S*. *pneumoniae* urinary antigen test, i.e. 6.71 (range 3.35–9.50) and 6.99 (range 1.79–8.67) log_10_ DNA copies/mL, respectively. Three patients with pneumococcal DNA detected had positive *S*. *pneumoniae* urinary antigen test but negative cultures from blood and respiratory secretions. Their NP pneumococcal densities were 7.76, 5.19, and 3.35 log_10_ DNA copies/mL.

Table 2 shows NP pneumococcal densities for different clinical and microbiological factors in 57 patients with pneumococcal pneumonia. In the univariate analysis, PSI risk class IV-V, admission to the intensive care unit (ICU), and a medium/high S-Ig titer to the patient´s own pneumococcal serotype, were associated with a high NP pneumococcal density (*p*<0.05). NP pneumococcal density was not associated with sex, age, smoking, co-morbidity, viral co-infection, pneumococcal serotype, or bacteremia.

**Table 2:** Clinical and microbiological factors and their association with nasopharyngeal pneumococcal density. Univariate analysis on 57 patients if not otherwise stated.

Factor		log_10_ DNA copies/mL		
	No. *with* factor/No. *without* factor	*With* factor, median pneumococcal density (interquartile range)	*Without* factor, median pneumococcal density (interquartile range)	*P-* value
Female gender	27/30	7.05 (6.22–7.95)	6.72 (5.59–7.66)	0.35
Age ≥65 years	34/23	6.77 (5.78–7.66)	6.86 (6.29–7.74)	0.49
Co-morbidity ^a^	33/24	6.71 (6.09–7.66)	6.84 (6.14–7.86)	0.72
Smoking, current or previous	35/22	6.83 (6.22–7.67)	6.79 (5.95–7.76)	0.83
Viral co-infection	14/43	7.35 (6.62–7.76)	6.71 (5.59–7.67)	0.31
Pneumonia severity index risk class IV-V	23/34	7.47 (6.24–7.97)	6.58 (5.95–7.56)	**0.04**
Symptoms ≥2 days prior to admission	37/20	7.05 (6.29–7.71)	6.26 (5.46–7.70)	0.10
Respiratory rate ≥ 30	20/37	7.22 (6.28–8.10)	6.66 (5.95–7.65)	0.12
Positive blood culture	12/45	6.94 (6.02–8.10)	6.73 (6.22–7.66)	0.61
Positive urine antigen test	33/24	6.71 (5.78–7.67)	6.99 (6.30–7.73)	0.56
Bilateral chest X-ray infiltrates	16/41	7.36 (6.70–8.00)	6.62 (6.07–7.66)	0.10
Admission to intensive care unit	3/54	8.06 (7.67–9.50)	6.72 (6.07–7.66)	**0.03**
Serotype with high degree of encapsulation ^b^	26/23	7.47 (6.42–7.79)	6.66 (6.22–7.97)	0.31
Medium/high S-Ig titer to the patient´s own serotype	30/17	7.61 (6.39–8.06)	6.66 (5.71–7.20)	**0.008**

^a^ Solid tumor, blood malignancy, liver disease, renal disease, chronic obstructive pulmonary disease, heart disease, stroke, diabetes.

^b^ Serotypes 3, 6B, 11A, 12F, 19A, 19F, 23F, and 35B.

To identify factors independently associated with a high NP pneumococcal density, we performed a regression analysis with bootstrapping. The included parameters were those that had a *p*-value of ≤0.1 in the univariate analysis (Table 2). We excluded admission to ICU since only three patients fulfilled this criterium. The analysis was based on 47 patients with complete information about all included parameters. As noted in Table 3, the following factors were independently associated with a high NP pneumococcal density: PSI risk class IV-V, symptom duration ≥2 days prior to admission, and a medium/high S-Ig titer against the patient´s own pneumococcal serotype.

**Table 3:** Factors and their independent association with nasopharyngeal pneumococcal density. Regression analysis with bootstrapping in 47 patients with complete information about the included parameters.

Factor	Mean pneumococcal density difference (log_10_ DNA copies/mL) between cases *with* and *without* factor (95% confidence interval)	*P*-value
Pneumonia severity index risk class IV-V	0.74 (0.12–1.41)	**0.023**
Symptoms ≥2 days prior to admission	0.89 (-0.01–1.67)	**0.039**
Bilateral chest X-ray infiltrates	0.16 (-0.50–0.80)	0.620
Medium/high S-Ig titer to the patient´s own serotype	1.03 (0.14–1.82)	**0.015**

### Performance of quantitative PCR for detection of severe pneumococcal pneumonia

Table 4 shows sensitivities and positive predictive values (PPV) at different NP DNA cut-off levels, for detection of severe pneumococcal pneumonia (PSI risk class IV-V). At different cut-off levels, sensitivities of 54–82% and PPV of 37–56% were noted.

**Table 4:** Sensitivities and positive predictive values for detection of severe pneumococcal pneumonia (Pneumonia Severity Index risk class IV-V) at different nasopharyngeal DNA density cut-off levels.

Cut-off(log_10_ copies/mL)	Sensitivity ^a^	Positive predictive value^b^
4.0	82 (23/28)	37 (23/63)
5.0	82 (23/28)	40 (23/57)
6.0	68 (19/28)	40 (19/47)
7.0	54 (15/28)	56 (15/27)

^a^ Data presented as % (patients with severe pneumococcal pneumonia and DNA density ≥ cut-off/all patients with severe pneumococcal pneumonia).

^b^ Data presented as % (patients with severe pneumococcal pneumonia and DNA density ≥ cut-off /all patients with DNA density ≥ cut-off).

## Discussion

This study on adult patients with pneumococcal pneumonia showed that symptom duration ≥2 days, a medium/high S-Ig titer against the patient´s own pneumococcal serotype, and pneumonia severity were independently associated with a high NP pneumococcal density.

To our knowledge, the association between pneumonia symptom duration and NP pneumococcal density has not been studied previously. In the pathogenesis of pneumococcal pneumonia, patients are usually colonized with *S*. *pneumoniae* in the nasopharynx, prior to micro-aspiration of NP secretions and subsequent development of pneumonia [7]. A long time of proliferation of bacteria in the nasopharynx can probably explain the association between a high NP pneumococcal density and long symptom duration.

Interestingly, a high NP pneumococcal density was independently associated with a medium/high S-Ig titer against the patient´s own pneumococcal serotype. The fact that many patients with short symptom duration had high S-Ig titers and that S-Ig titers were not correlated to symptom duration, indicates that serotype-specific antibodies often developed during the pre-pneumonia colonization period. Previous studies have shown that pneumococcal colonization often induces a serotype-specific S-Ig response [7, 24, 25]. Our group recently reported that asymptomatic controls colonized with *S*. *pneumoniae* had a median S-Ig titer (101 AU; range, 15–225 AU) at hospital admission, similar to that of patients with non-bacteremic pneumococcal pneumonia (94 AU; range, 2–243 AU), but higher than that of patients with bacteremic pneumococcal pneumonia (24 AU; range, 0–112 AU) [18]. The median S-Ig titer was significantly lower for cases with bacteremic pneumococcal pneumonia than for cases with non-bacteremic pneumococcal pneumonia (*p* = 0.043) [18]. This suggests that S-Ig may be protective against subsequent bacteremia in patients with pneumococcal pneumonia. Recently, when Ferreira et al. [25] injected serum samples from healthy individuals with experimental NP colonization into mice, the mice were protected against pneumococcal infection at challenge with lethal doses of *S*. *pneumoniae*. Thus, our hypothesis is that NP colonization will induce development of serotype-specific S-Ig, which protects against proliferation of the colonizing *S*. *pneumoniae* to pneumonia and/or to invasive pneumococcal disease. However, after S-Ig has developed during colonization, *S*. *pneumoniae* of the colonizing serotype can probably still cause pneumonia, if the density of *S*. *pneumoniae* in the nasopharynx is high.

In agreement with the study by Albrich et al. [10], the present study showed an association between a high NP pneumococcal density and disease severity. The reason for this association has not been clarified. A high NP pneumococcal density may be a preceding cause of severe pneumonia. However, a more likely explanation is that a high NP pneumococcal density is a subsequent effect of having severe pneumonia. In patients with severe pneumococcal pneumonia, high concentrations of bacteria are often generated in the lower respiratory tract, and secretions with high loads of pneumococcal DNA may end up in nasopharynx during coughing. A high NP pneumococcal density may thus be an indicator of a high pneumococcal load throughout the respiratory tract. The pneumococcal load in the bloodstream has clearly been linked to disease severity [26, 27]. However, the association between pneumococcal load in the lower respiratory tract and disease severity has not been properly studied, to our knowledge. In a population of CAP patients who were current/previous smokers, Werno et al. [27] found that a high pneumococcal DNA load (>10^5^colony-forming units/mL) in sputum was associated with severe disease based on PSI (odds ratio 2.73; 95% confidence interval, 1.01–7.36).

The association between NP pneumococcal density and pneumonia severity in the present study encouraged us to study if NP pneumococcal density could be used for detection of severe pneumococcal pneumonia. Our evaluation showed sensitivities of 54–82% and PPV of 37–56% (Table 4), which indicates that quantitative PCR applied to NP aspirate has limited value for detection of severe pneumonia. However, PCR for detection of pneumococcal DNA in respiratory secretions remains a promising method for rapid establishment of pneumococcal etiology, in populations with low frequency of pneumococcal colonization [23].

Viral co-infection has previously been associated with a high NP pneumococcal density [28, 29]. In Vietnamese children with X-ray verified pneumonia [28], subjects with viral co-infection had a 15-fold higher *S*. *pneumoniae* NP load than subjects without viral co-infection. Thus, we were surprised that we could not find any clear correlation between NP pneumococcal density and viral co-infection in the present study, even though viral co-infection was noted in 25% of the patients (Table 2). The difference is probably due to the different loads of *S*. *pneumoniae* in the nasopharynx of children and adults [7, 29]. Significant *S*. *pneumoniae* NP loads are often noted in healthy children [28], although only 3.6% of asymptomatic adults had an NP pneumococcal density of ≥ 10^4^ DNA copies/mL in our previous study [9]. Another possible explanation for the difference between our study and the pediatric study [28] may be that we used a serological method for detection of viral infection, although the other group used a PCR method.

A very clear finding in the present study was that the frequency of pneumococcal etiology and presence of pneumococcal DNA in the nasopharynx were low in CAP patients who had received antibiotics prior to admission (Table 1). We believe that this can mainly be explained by the fact that the recommended outpatient CAP therapy in Sweden is focused on *S*. *pneumoniae* [30]. The outpatient therapy will probably cure the majority of outpatients with pneumococcal pneumonia, although outpatients with CAP caused by other etiologies will not be cured as often and may require hospitalization.

The present study has some limitations. First, as the number of study subjects was relatively small, we could not properly evaluate if NP pneumococcal density differs between pneumococcal serotypes or if it correlates to mortality. Secondly, ELISA serology does not provide any information about antibody function, which can be provided by opsonophagocytic serology. A similar study with opsonophagocytic serology would provide additional knowledge.

The S-Ig ELISA method of the present study was used as a standard routine test prior to presentation and introduction of the ELISA method described by Wernette et al. [22], which has since become an established method for evaluation of polysaccharide antibodies after pneumococcal vaccination. As our serum samples were collected and tested for antibody titer before the method paper of Wernette et al. was published in 2003, we were not able to use that method in the present study. However, the ELISA method used in this paper is very similar to the method of Wernette at al., as we have previously described [31], although the results are presented as AU instead of μg/ml. Thus, we think that this method was appropriate for the present study. Data from the present ELISA method has previously been presented in another study for evaluation of antibody response after pneumococcal infection [18].

## Conclusions

The study showed that NP pneumococcal density was associated with pneumonia severity in an unselected pneumococcal pneumonia population. However, NP pneumococcal density was found to have limited value for detection of severe pneumococcal pneumonia. The study showed, for the first time to our knowledge, that symptom duration ≥2 days and that a medium/high S-Ig titer against the patient´s own pneumococcal serotype were independently associated with a high NP pneumococcal density. However S-Ig titers were not associated with symptom duration and were often high in patients with short symptom duration. This indicates that serotype-specific Ig often developed during the pre-pneumonia colonization period. The association between high S-Ig titer and high NP pneumococcal density suggests that in patients with high S-Ig titers induced by colonization, a high NP pneumococcal density is often required for development of pneumococcal pneumonia.
